# Preparation of Green Tea Polyphenol-Loaded Diacylglycerol Nanostructured Lipid Carrier Hydrogels and Their Activities Related to Skin Protection

**DOI:** 10.3390/ma17246227

**Published:** 2024-12-20

**Authors:** Zhini Zhu, Qiu Xia, Xinxia Zhan, Wenyuan Li, Xuan He, Bo Wang, Qizhi Zhou, Jian Huang, Yong Ye

**Affiliations:** 1 School of Chemistry and Chemical Engineering, South China University of Technology, Guangzhou 510640, China; zzn18727377565@163.com (Z.Z.); xq19834534364@163.com (Q.X.); 18319811698@163.com (X.Z.); 19114787694@163.com (W.L.); 2 Ganzhou Hake Biotech Co., Ltd., Ganzhou 341008, China; 15970079778@163.com; 3 Ganzhou Forestry Science Research Institute, Ganzhou 341000, China; 13879729572@163.com; 4 Hunan Singular Biotech Co., Ltd., Changsha 410329, China; 5 Jiangxi Ruijia Biotech Co., Ltd., Yichun 330899, China

**Keywords:** diacylglycerol, lipid carriers, hydrogel, skin protection

## Abstract

Diacylglycerol (DAG) is a functional oil but is rarely used in the cosmetic industry because low solubility, susceptibility to leakage and low viscosity to skin are still the main hurdles. A novel diacylglycerol nanostructured lipid carrier hydrogel (GTP-DAG-NLC-GEL) loaded with green tea polyphenol (GTP) was designed and successfully prepared to broaden DAG’s application in cosmetics, which significantly improved GTP stability and skin stickiness of DAG. The results showed that DAG-NLC-GEL had good viscosity, which was 980 Pa·s when the shear rate was 5 rpm, and its viscosity decreased quickly with the increase in shear rate, making it easily expand on skin. Meanwhile, the encapsulation rate and drug loading of GTP in GDP-DAG-NLC-GEL reached 86.7% and 2.6%, respectively, and the DPPH free radicals scavenging rate and inhibition rate of the advanced glycation end-products (AGEs) were 85.46% and 89.72%, respectively, which indicate that GTP-DAG-NLC-GEL has significant skin sunscreen, antioxidant and anti-glycation activities. The GTP-loaded nanostructured lipid carrier hydrogel can be deemed to have great prospects for skin protection in cosmetics.

## 1. Introduction

The skin serves as the body’s principal protective barrier against environmental damage. Nonetheless, oxidative stress and glycation are common factors that can adversely affect the skin, thereby accelerating the aging process [1,2]. Green tea polyphenols (GTPs) possess phenolic hydroxyl groups with antioxidant activities, enabling them to act as potent free radical scavengers. This property facilitates the elimination of harmful free radicals from the body and prevents cell membrane damage, thereby mitigating skin aging [3]. Additionally, GTP exhibits ultraviolet (UV) absorption and enzyme inhibition properties, rendering them suitable for applications in skin whitening and sunscreen products and making them a valuable natural resource for cosmetic formulations [4]. However, GTP is notably unstable in the use environment and easily oxidized by air [5,6].

Nanostructured lipid carriers (NLCs) represent an innovative drug delivery system characterized by superior encapsulation efficiency and biocompatibility, alongside enhanced therapeutic efficacy and skin hydration, in comparison to solid lipid nanoparticles (SLNs) [7,8,9]. These attributes make NLCs promising candidates for dermatological and cosmetic applications [10]. Keck et al. developed a silver–NLC formulation aimed at treating atopic dermatitis [11]. Siriporn et al. studied the occlusive qualities and antioxidant activity of lycopene-loaded NLCs. But their results suggested that NLCs had limited enclosure properties and weak skin hydration [12]. To overcome the problem that NLCs have low viscosity and are not suitable for skin application, they can be gelled or mixed into semi-solid systems to improve the consistency of the final formulation. However, the mixture is unstable, and new NLCs with excellent enclosure properties and skin hydration need to be investigated.

Triglycerides serve as a crucial lipid substrate in the formulation of nanostructured lipid carriers (NLCs). However, their pronounced hydrophobic nature impedes the effective encapsulation of hydrophilic pharmaceuticals, thereby restricting their utility in cosmetics. In contrast, diacylglycerols (DAGs) exhibit enhanced amphiphilic properties, which facilitate the incorporation and delivery of drug compounds more effectively than triacylglycerols. Feng demonstrated that a DAG oil-based nanoemulsion delivery system held significant potential for enhancing emulsification properties and augmenting the biological activity of pharmaceutical products relative to TAG [13]. Similarly, Liu reported that the solubility of various hydrophobic cyclic peptide drugs in DAG was nearly 20 times greater than in other substrates, indicating that DAG can effectively enhance emulsion stability and bioavailability [14]. Furthermore, diacylglycerol has been found to enhance skin hydration and address various skin disorders. Given these beneficial properties, diacylglycerol holds significant potential for applications in the cosmetic industry [15]. Nonetheless, there is a lack of literature on the utilization of diacylglycerol as a carrier in cosmetic formulations.

In order to improve the stability of tea polyphenols and open the application of diacylglycerol on cosmetics, a novel NLC based on diacylglycerol is designed to load GTP, and the NLC hydrogels are prepared to strengthen the viscosity to improve suitability for skin application. Their physical properties including particle size, zeta potential, polydispersity index (PDI) and morphology are determined, and their skin protective effects such as anti-glycation, sunscreen and antioxidant activities are further evaluated.

## 2. Materials and Methods

### 2.1. Materials

GTP (total polyphenols content ≥ 95 g/100 g, total catechins content ≥ 80 g/100 g, epicallocatechin-3-gallate (EGCG) content ≥ 50 g/100 g), N-Octanoic acid, hexane, soy lecithin, 90% tea saponin, imidazolidinyl urea, carbomer 940, triethanolamine, dimethyldiarbazide hydrochloride. *Camellia* oil was purchased from Shanghai McLean Biochemistry & Science Technology Co, Ltd. (Shanghai, China). All compounds were of analytical purity and could be utilized without additional purification.

### 2.2. Preparation of Diacylglycerol NLC

The preparation of diacylglycerol NLC was referred to the reference with minor modifications, in which *Camellia* oil was mixed with diacylglycerol in the weight ratio of 2:3 as the oil phase [16]. Soy lecithin (2% *w*/*w*), tea saponin (0.5% *w*/*w*) and glycerol (4% *w*/*w*) were used as the aqueous phase, which was added to oil phase at 75 °C, emulsified for 3 min at 1200 r/min, sonicated for 15 min at 70% and then cooled down to room temperature to obtain the NLC.

### 2.3. Preparation of GTP-DAG-NLC-GEL

Based on the preliminary pre-screening, combined with the stability of the material and the performance of sunscreen, antioxidant and anti-saccharification (Figure S1), octanoic acid diglyceride was selected as the raw material for the preparation of DAG-NLC. Similar to the preparation method mentioned above, GTP was added to the water phase as the encapsulated drug. In order to produce the GTP-DAG-NLC, the aqueous phase was added to the oil phase for preliminary emulsification, and the primary emulsion was homogenized using a homogenizer (T18, Guangzhou Huaqiao Instrument Technology Co., Ltd., Guangzhou, China) at a speed of 23,000 r/min for 10 min. The primary emulsion was dispersed through an ultrasonic cell crusher (JY92-11N, Shanghai HUANYAN Industrial Co., Ltd., Shanghai, China) while it was still hot and cooled to room temperature in an ice water bath.

One gram of carbomer 940 was weighed and mixed with 100 g of 10% glycerol aqueous solution. The mixture was thoroughly stirred until the carbomer 940 was fully dispersed in the water. Triethanolamine was then added to the solution in a 1:1 volume ratio relative to the carbomer. Given the small volume of carbomer 940 (1 g), an equivalent volume of triethanolamine (1 g) was required. Stirring was continued until the pH of the solution reached neutrality (approximately pH 7), with adjustments to the amount of triethanolamine as necessary. The hydrogel was successfully synthesized when the carboxyl groups of the carbomer were adequately neutralized by the triethanolamine. GTP-DAG-NLC-GEL was created by adding the mentioned GTP-DAG-NLC at 1:1 mass ratio to the hydrogel and mixing it for 10 min at 300 rpm. After stirring, the homogeneity of the mixture was checked to make sure that there was no aggregation or precipitation of GTP-DAG-NLC, and GTP-DAG-NLC-GEL was made after confirming the homogeneity of the mixture.

### 2.4. Physicochemical Characterization

#### 2.4.1. Particle Size, PDI and Zeta Potential

The samples were diluted with distilled water to a suitable concentration until the liquid was clear. Average size, PDI and zeta potential of the samples were measured with Malvern Instruments (Malvern Instruments, Malvern, UK). Three parallel experiments were performed with the following conditions: particle refractive index 1.4, particle absorption index 0.001 and solvent refractive index 1.33. The average of the three measurements was then calculated.

#### 2.4.2. Morphological Observation

Transmission electron microscope (JEM2100, Electronics Co., Ltd., Akishima, Japan) was used to observe the morphology of the samples. Appropriate amount of sample was diluted with distilled water, mixed homogeneously by ultrasonication and added dropwise on the copper mesh. Transmission electron microscopy was used to view the samples after the copper mesh was allowed to dry at room temperature in a fume chamber.

#### 2.4.3. X-Ray Diffraction Analysis

A significant quantity of the freeze-dried GTP-DAG-NLC-GEL was put on a slide and compacted for XRD analysis (D/MAX-IIIA, Rigaku Co., Ltd., Akishima, Japan) after processing. Testing conditions: Cu target Cu Kα (λ = 0.1540598 nm), test tube pressure 40 kV, test tube current 40 mA, scanning range (2θ) 5°–60°, scanning rate 2°/min, step size 0.013°.

#### 2.4.4. FT-IR Spectra

The infrared spectra of GTP and GTP-DAG-NLC-GEL were scanned using a Fourier transform infrared spectrometer (Tensor, Beijing, China). Using a tablet press, the GTP and lyophilized samples of GTP-DAG-NLC-GEL were combined 1:100 with potassium bromide, respectively. Infrared spectroscopic scans of the pressed samples were performed in the 500–4000 cm^−1^ range.

#### 2.4.5. Thermal Stability

The rate of weight loss of GTP and GTP-DAG-NLC-GEL lyophilized samples was determined using a thermogravimetry instrument (TGA550, Discovery, New York, NY, USA), and the data were recorded over a scanning range of 0–550 °C.

#### 2.4.6. Differential Scanning Calorimetry (DSC)

The thermodynamic properties of DAG-NLC and GTP-DAG-NLC were measured using a Differential Scanning Calorimeter (DSC250, PerkinElmer, Waltham, MA, USA). The samples (2–3 mg) sealed in an aluminum pot were heated from −30 °C to 90 °C at a constant heating rate of 5 °C/min with a nitrogen flow rate of 20 mL/min.

#### 2.4.7. Viscosity Test

To investigate the flow behavior of the hydrogels, a rotating viscometer (NDJ-1, Shanghai Li-Chen Bang Xi Instrument Technology Co., Ltd., Shanghai, China) was used to perform continuous shear experiments at room temperature. The viscosity of GTP-DAG-NLC hydrogels was assessed at 60 rpm to compute the relationship between shear rate (rpm) and shear stress (Pa·s).

#### 2.4.8. Stability Analysis

Storage stability: Particle size, PDI value and zeta potential of GTP-DAG-NLC-GEL were determined under various storage circumstances after hydrogel was stored for 5 days, 10 days and 15 days at varying temperatures (0–5 °C, 25 °C) [17].

Centrifugal stability: A centrifuge tube containing 1 g of GTP-DAG-NLC-GEL was weighed and centrifuged for 30 min at 12,000 r/min. The stability parameter *K*_E_ can be used to express the centrifugal stability.

(1) $$K_{E}=\frac{|R_{0}-R|}{R_{0}}$$

where *R*_0_ denotes the particle size of hydrogel before centrifugation and *R* denotes the particle size of hydrogel after centrifugation.

#### 2.4.9. Loading Capacity and Encapsulation Efficiency

GTP-DAG-NLC-GEL lyophilized sample (20 mg) was added to 10 mL ethanol solution (99%) for ultrasonic treatment for 10 min so that tea polyphenols were completely released in the solution. An amount of 0.1 mL of supernatant solution was aspirated and the volume was adjusted with ethanol to 10 mL to obtain the test solution. The lyophilized DAG-NLC-GEL sample was handled identically to be a blank sample. A UV spectrophotometer (UV2450, Shimadzu, Kyoto, Japan) was used to measure the absorbance of the supernatant at 274 nm after it was transferred to a quartz cuvette. According to the tea polyphenol standard curves, the concentration of tea polyphenol in the samples was determined. Equations (2) and (3) were used to determine the drug loading (*γ*) and encapsulation rate (*η*) [18]:

(2) $$\gamma =\frac{m_{t}}{m_{n}}\times 100\%$$

(3) $$\eta =\frac{m_{t}}{m_{at}}\times 100\%$$

where *m*_t_ is the mass of GTP in the GTP-DAG-NLC-GEL, *m*_n_ is the mass of the hydrogel itself and *m*_at_ is the mass of the actual input GTP.

#### 2.4.10. In Vitro Release Rate

According to the previous method for determining the in vitro release rate of GTP-DAG-NLC-GEL, the samples were weighed and dispersed in PBS buffer solution (pH = 7.5), dialyzed in a dialysis bag with cutoff of a relative molecular mass of 14,000 and shaken in a thermostatic water bath (100 r/min, 37 °C). An amount of 10 mL of each solution was collected at various time intervals and refilled with an equal volume of new medium. The absorbance of the samples was determined at 274 nm using a UV spectrophotometer (UV2450, Shimadzu, Japan) to plot the kinetic curves of GTP vs. time release. The release velocity (*θ*) was estimated using Equation (4):

(4) $$\theta =\frac{m_{s}}{m}\times 100\%$$

where *m*_s_ is the cumulative drug release at a time point and *m* is the total content of drug in the GTP-DAG-NLC-GEL.

### 2.5. Skin Protection Tests

#### 2.5.1. Sun Protection Performance Test

An amount of 0.1 g of sample was dissolved in 200 mL water and scanned using a UV spectrophotometer (UV2450, Shimadzu, Japan) at a wavelength of 200–400 nm.

#### 2.5.2. Antioxidant Activity Test

Free radical scavenging (antioxidant) capacity was measured by 1,1-diphenyl-2-trinitrohydrazine (DPPH) assay. After mixing 2.0 mL of sample solution with 2.0 mL of DPPH ethanol solution at a concentration of 2 mmol/L, the mixture was thoroughly shaken. A UV spectrophotometer (UV2450, Shimadzu, Japan) was used to determine the absorbance A_i_ at 517 nm after the combined samples were incubated for 30 min at 37 °C. The absorbance at 517 nm of the sample solution after it had been thoroughly mixed with 2.0 mL of anhydrous ethanol was denoted by A_j_. The absorbance at 517 nm of the DPPH ethanol solution after it had been thoroughly mixed with 2.0 mL of anhydrous ethanol is *A*_0_. The data were presented as mean ± standard deviation and all calculations were made in triplicate. The following Formula (5) was used to determine the DPPH radical scavenging rate for each sample:

(5) $$Scavengingactivity\left(\%\right)=\left(\frac{1-\left(A_{i}-A_{j}\right)}{A_{0}}\right)\times 100\%$$

where *A*_0_ is the absorbance of the blank group; *A*_i_ is the absorbance of the experimental group; and *A*_j_ is the absorbance of the sample solution.

#### 2.5.3. Anti-Glycation Test

The anti-glycation test was carried out using the previously reported method, which involved creating an in vitro glycation reaction system with bovine serum protein and glucose to replicate the human environment [19,20]. Under relatively aseptic conditions, a glycation system with bovine serum albumin solution of 20 g/L and glucose solution of 400 mmol/L was used. The groups were set up as follows: (1) a complete glycation reaction system without addition of the test solution; (2) a control group containing only bovine serum albumin; (3) a glycation system with addition of the test solution and no addition of bovine serum albumin; (4) a glycation system with addition of the test solution; (5) a drug group plus the whole glycation reaction system of the test solution.

The reaction systems were incubated away from light for 40 days. The fluorescence intensity of each group of reaction solution was measured using a fluorescence spectrophotometer (UV2450, Shimadzu, Japan) with an excitation wavelength of 370 nm, emission wavelength of 440 nm and slit of 5 nm. The inhibition rates of the advanced glycation end-products (AGEs) were determined using the following Formula (6):

(6) $$I=\frac{1-\left(F_{5}-F_{4}-F_{3}\right)}{F_{1}-F_{2}}\times 100\%$$

where *F*_1_, *F*_2_, *F*_3_, *F*_4_, and *F*_5_ represent the fluorescence intensities of the reaction solutions in groups 1, 2, 3, 4, and 5, respectively.

### 2.6. Statistical Analyses

All experiments were carried out in triplicate and then the results were presented as mean value ± standard deviation. The analyses of data and the depiction of the figures were performed by using OriginPro2018 (Origin Lab Corporation, Northampton, MA, USA) software with one-way analysis of variance (ANOVA). Tukey’s test was used to analyze the differences (*p* < 0.05).

## 3. Results

### 3.1. Morphology and Particle Size of DAG-NLC

**Figure 1:**
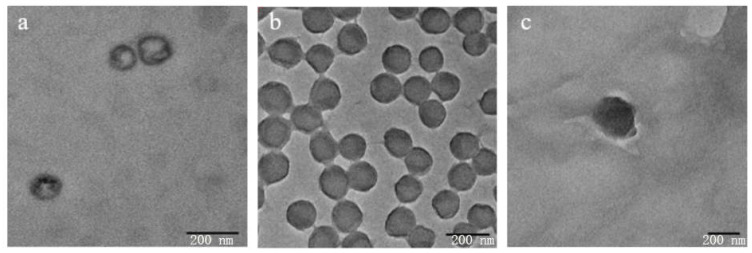
TEM images of (**a**) DAG-NLC, (**b**) GTP-DAG-NLC, (**c**) GTP-DAG-NLC-GEL.

As shown in Figure 1a, DAG-NLC had a uniform spherical structure with a smooth surface and existed in the form of α crystals of the traditional lipid phase [21]. The material morphology was basically unchanged after loading GTP (Figure 1b), suggesting that the load of GTP has no effect on the material morphology and structure. Because α crystals existed in a defect state, which could provide more space for drug loading, the particle size and PDI of GTP-DAG-NLC-GEL were similar to DAG-NLC-GEL, but the zeta potential was significantly reduced (Table 1). As demonstrated in Table 2, while the incorporation of hydrogel resulted in an increased particle size of GTP-DAG-NLC, its dispersion and stability were better than most of the previous tea polyphenol-loaded nano-lipid carriers [22,23,24,25,26,27], and it had a better loading capacity and stability. Meanwhile, the GTP-TAG-NLC-GEL network can be clearly observed in Figure 1c, suggesting that the semi-solid formulation developed in this study is effective for the combination of these systems and GTP-loaded hydrogels with higher stability.

### 3.2. Spectra Characterization of GTP-DAG-NLC

**Figure 2:**
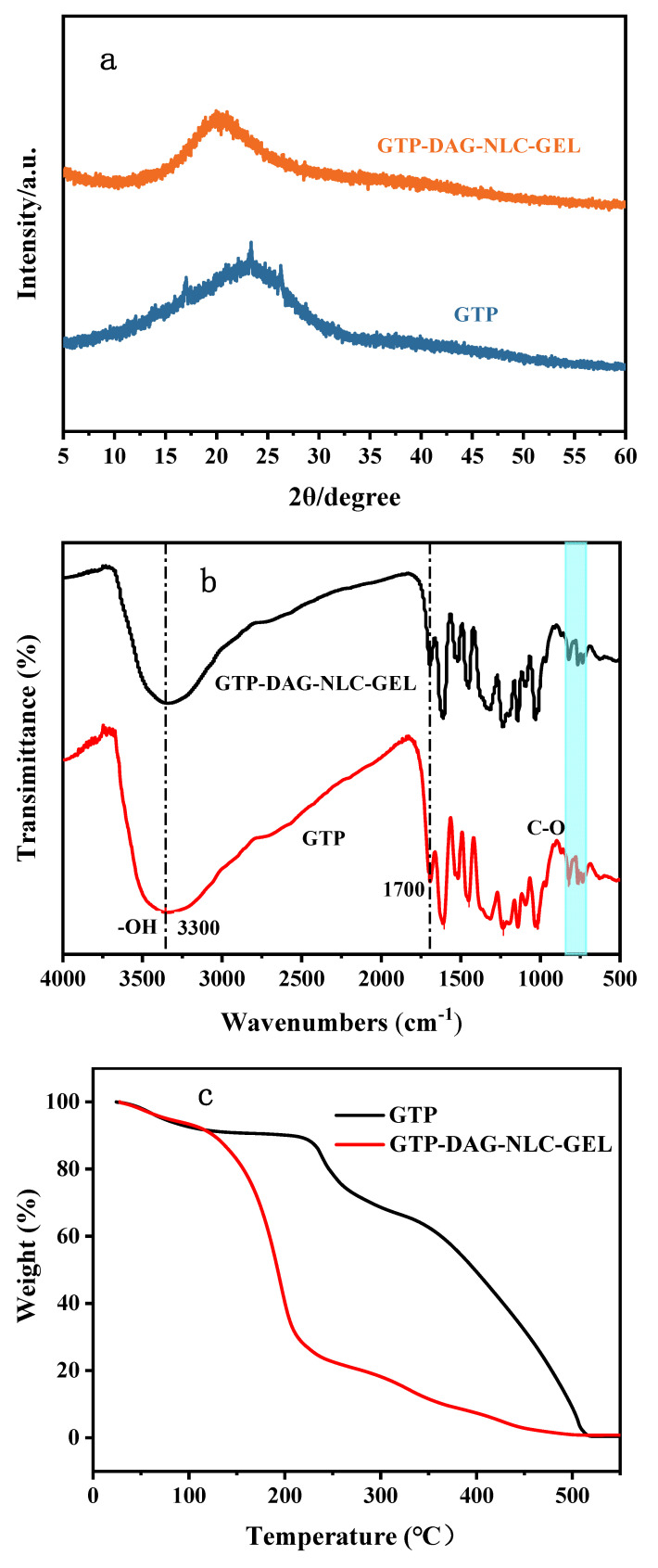

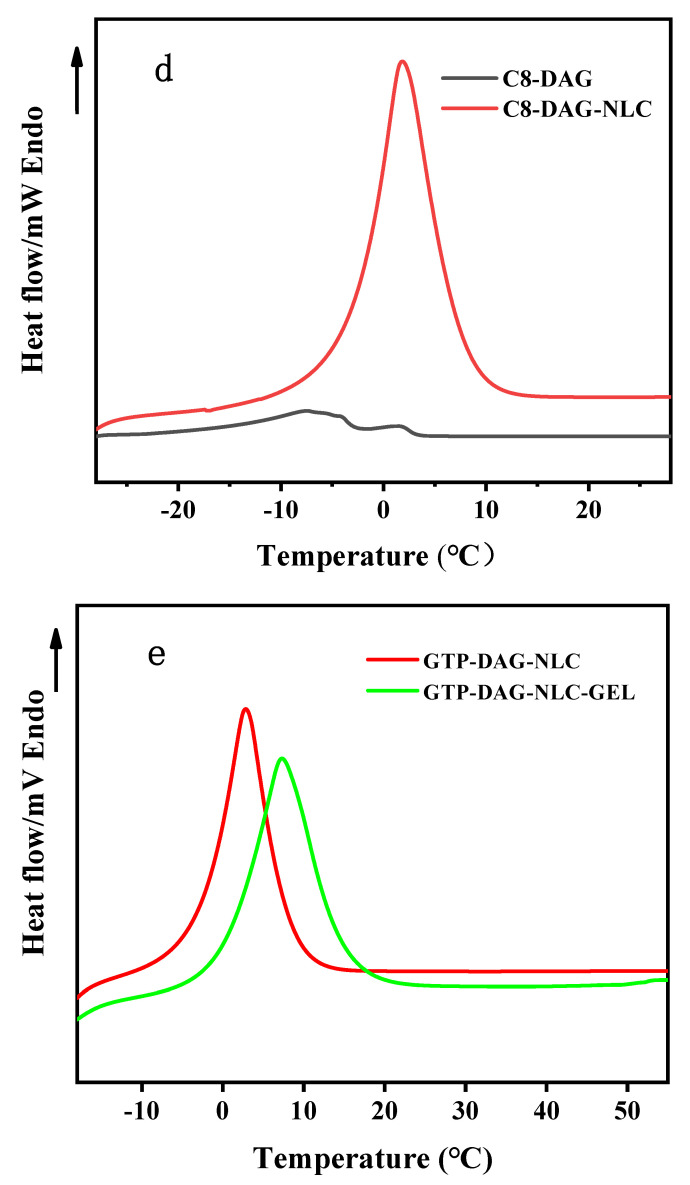
(**a**) XRD pattern of GTP-DAG-NLC-GEL and GTP. (**b**) FT-IR spectra of GTP-DAG-NLC-GEL and GTP. (**c**) Thermogravimetric curves of GTP-DAG-NLC-GEL and GTP. (**d**) DSC curves of octanoic acid diglyceride and its DAG-NLC. (**e**) DSC curves of GTP-DAG-NLC and GTP-DAG-NLC-GEL.

The XRD pattern showed that the characteristic peaks of GTP-DAG-NLC-GEL became wider and shorter, and the peak strength at 16.3 Å, 23.1 Å and 25.8 Å decreased significantly in Figure 2a, indicating that the GTP crystal order in GTP-DAG-NLC-GEL is decreased [28]. However, it still maintained a certain degree of crystallinity, confirming that GTP is successfully loaded in NLC hydrogel. To provide additional proof, FT-IR analysis was carried out. As depicted in Figure 2b, the characteristic peaks at 3300 cm^−1^, 1700 cm^−1^ and 700 cm^−1^ correspond to the stretching vibrations of O-H, C=C/C=O and C-O, respectively. Meanwhile, these characteristic peaks could be observed in FT-IR spectra of GTP-DAG-NLC but the peak intensity declined significantly. This suggests that the preparation of GTP-DAG-NLC-GEL was successful.

The thermal stability curves of GTP-DAG-NLC-GEL and GTP are shown in Figure 2c. The thermogravimetric process of GTP could be divided into three stages: The first stage was from room temperature to 220 °C, during which the mass of GTP decreased slightly due to the loss of bound water, and the weight loss rate was about 10%. In the second stage from 220 °C to 350 °C, the mass of GTP dropped sharply due to the oxidation of hydroxyl groups, and the weight loss was about 31%. In the third stage, when the temperature exceeded 350 °C, the mass rapidly decreased to 0 because of main block oxidation, which was in accord with the literature reports. The weight loss rate of GTP-DAG-NLC-GEL could also be divided into three stages. In the first stage, the weight loss was lower than that of GTP from room temperature (25 °C) to 100 °C, suggesting that GTP-DAG-NLC-GEL has moisturizing properties. The second stage was 100 °C to 220 °C, this stage might be due to the rapid evaporation of water in the hydrogel, the weight loss was 83%. In the third stage, the weight loss rate began to slow down when the temperature was above 220 °C, which might be due to the diacylglycerol encapsulating GTP and reducing its oxidation. This indicates that GTP-DAG-NLC-GEL has good thermal stability.

As demonstrated in Figure 2d, DAG-NLC had only one endothermic peak, indicating the presence of crystals with stable structures in its interior. At the same time, the intensity of the endothermic peak of the NLC was greater than that of DAG and the position of the peak was redshifted, indicating that a new phase is formed due to the successful preparation of DAG-NLC. In addition, the melting point of the NLC and DAG was inconsistent. This difference suggests that during the preparation of the NLC, the combination of DAG and Camellia oil changes the crystalline structure of DAG to a certain extent, resulting in its crystallization and transformation into a defect state [8]. The heat absorption curves of GTP-DAG-NLC before and after the incorporation of the hydrogel are shown in Figure 2e. Both GTP-DAG-NLC and GTP-DAG-NLC-GEL had only one endothermic peak, indicating the existence of a stable crystalline structure. When the hydrogel was formed, the intensity of the endothermic peak became lower, suggesting that a new phase formed. At the same time, the melting point of GTP-DAG-NLC-GEL was higher than that of GTP-DAG-NLC, which might be due to the increase in particle size and the decrease in specific surface area after hydrogels coating the NLC, in line with the “Kelvin effect” [29].

### 3.3. Viscosity of GTP-DAG-NLC-GEL

**Figure 3:**
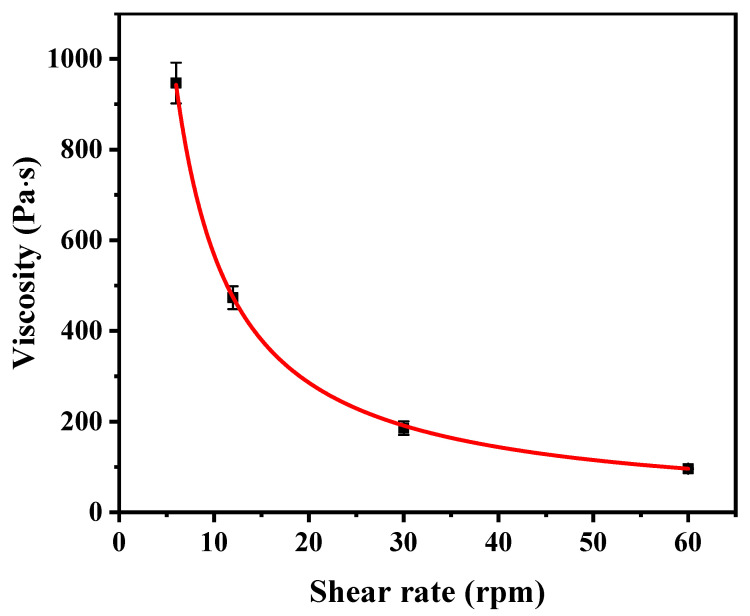
Viscosity of GTP-DAG-NLC-GEL at different shear rates.

As shown in Figure 3, the viscosity of GTP-DAG-NLC-GEL was 980 Pa·s at a shear rate of 5 rpm and decreased significantly with the increase in shear rate, demonstrating the characteristics of shear thinning, which were similar to those reported by Tichota [30]. The data indicate that the GTP-DAG-NLC-GEL is sticky to the skin and external friction makes it easy to expand on the skin.

### 3.4. Storage Stability of GTP-DAG-NLC-GEL

The hydrogel may degrade or accumulate during long-term storage, resulting in the release of embedded compounds. However, Table 3 lists the absolute value of the zeta potential of GTP-DAG-NLC-GEL, which was greater than 30 mV at different temperatures for up to 15 d of storage time, indicating that it is stable [31]. Meanwhile, the particle size of GTP-TAG-NLC-GEL increased gradually with the increase in time due to the decrease in interparticle repulsion corresponding to the exponential increase in PDI. It is worth noting that the change in particle size was smaller at 0–5 °C, indicating that lower temperatures are more conducive to the stability of GTP-DAG-NLC-GEL for long-term storage [32].

### 3.5. In Vitro Release of GTP

**Figure 4:**
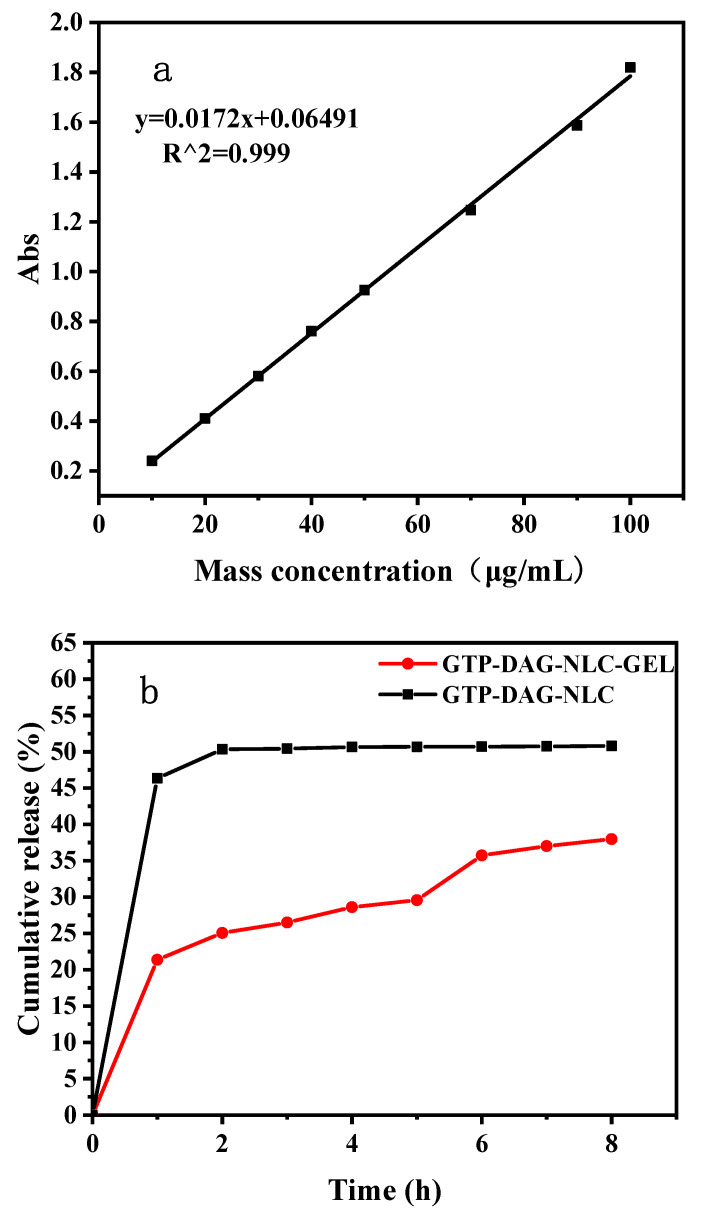
(**a**) Standard curve of absorbance vs. concentration of GTP. (**b**) The cumulative release curves of GTP-DAG-NLC and GTP-DAG-NLC-GEL.

In hydrogels, the encapsulation efficiency and drug loading rates of GTP were determined by a UV spectrophotometer, and the measured standard curves were illustrated in Figure 4a. Due to the high affinity of DAG-NLC for GTP, the encapsulation efficiency and drug loading rate of GTP in GTP-DAG-NLC-GEL reached 86.7% and 2.6%, respectively (the additional amount of GTP was 3%). It means that GTP-DAG-NLC-GEL is suitable as a carrier to transport GTP [33]. As shown in Figure 4b, the cumulative release curve of GTP in the GTP-DAG-NLC presented two stages of rapid release and slow release, while the GTP-DAG-NLC-GEL system presented three stages of release. The cumulative release rate of GTP-DAG-NLC was 50.80% within 8 h, which might be due to the rapid release of GTP adsorbed in the outer layer or the emulsion layer of NLC [34]. GTP-DAG-NLC-GEL had a similar release trend at the initial stage due to the rapid release of GTP-NLC on the hydrogel surface, but the cumulative release amount (37.98%) was lower than that of GTP-DAG-NLC. And with the extension of the release time, the release rate gradually slowed down. When the solvent penetrated the hydrogel, the GTP-DAG-NLC in the hydrogel was dispersed in the solvent, causing the rate to increase again. In summary, GTP-DAG-NLC reduces GTP release, and GTP-DAG-NLC-GEL can more effectively extend the release of GTP.

### 3.6. Skin Protection Activity of GTP-DAG-NLC-GEL

#### 3.6.1. Sun Protection Performance

**Figure 5:**
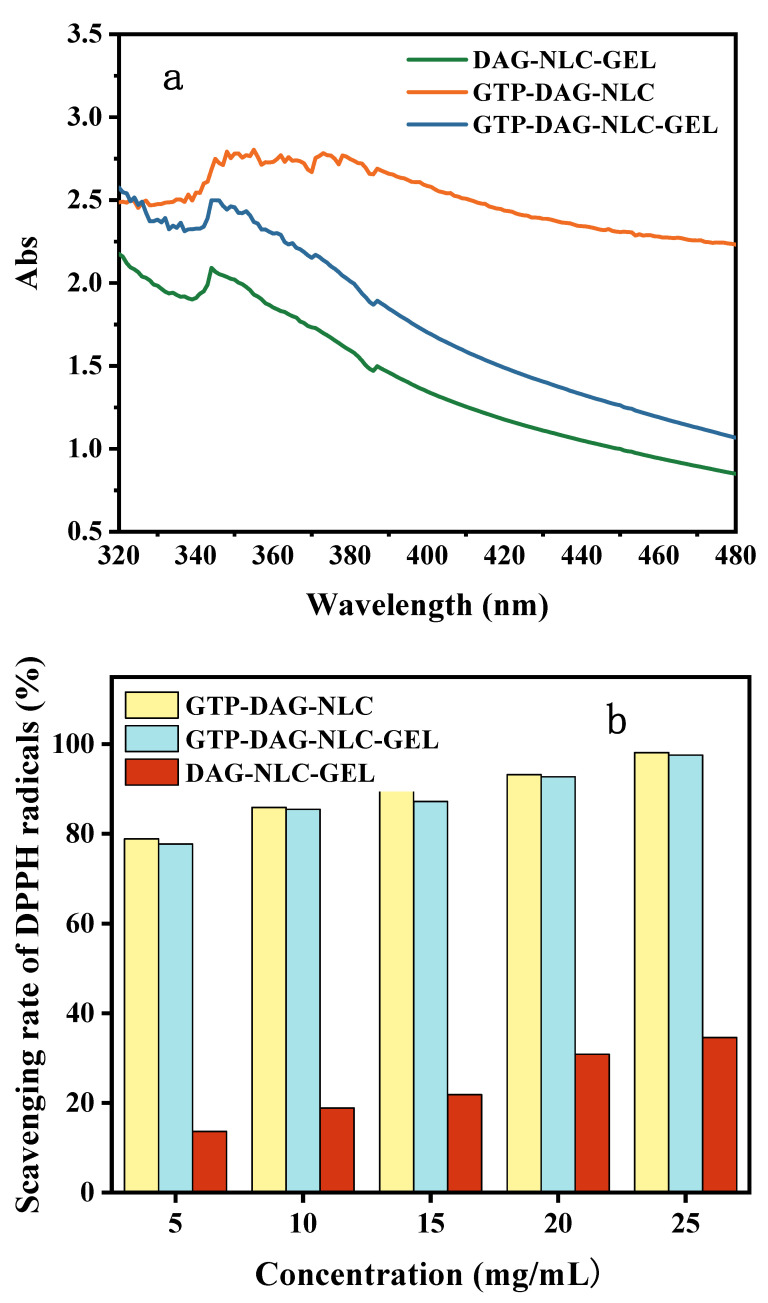

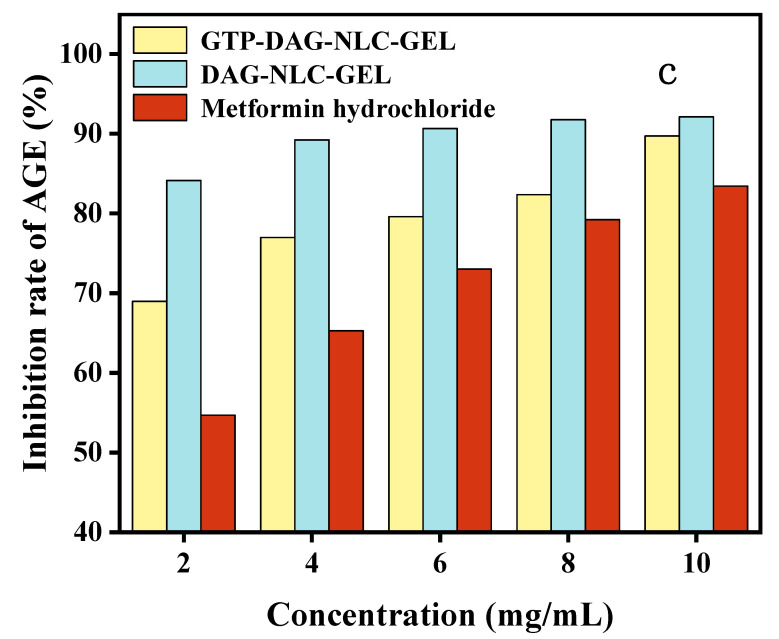
Skin protection activity of GTP-DAG-NLC-GEL: (**a**) UV-vis absorbance spectra. (**b**) DPPH free radical scavenging rate. (**c**) Inhibition rate of AGE.

The UV–visible absorbance of DAG-NLC-GEL, GTP-DAG-NLC and GTP-DAG-NLC-GEL were investigated to evaluate their UV-blocking effects. As shown in Figure 5a, the UV absorbance of GDP-DAG-NLC-GEL was between GTP-NLC-GEL and GTP-DAG-NLC in the wavelength range of UVA (320~400 nm) [35]. It is well known that GTP is a natural polyphenol that contains many chromophores such as phenol and keto groups that can absorb UV rays [36]. Therefore, the sunscreen performance of GTP-DAG-NLC-GEL was stronger than that of DAG-NLC-GEL. Combining the UV absorption ability of GTP with DAG-NLC, GTP-DAG-NLC had a strong sunscreen activity. However, the absorbance of the hydrogel itself was very low, resulting in the UV absorption of GTP-DAG-NLC-GEL being lower than that of GTP-DAG-NLC [37].

#### 3.6.2. Antioxidant Activity

The antioxidant properties of DAG-NLC-GEL, GTP-DAG-NLC and GTP-DAG-NLC-GEL were evaluated by a DPPH free radical scavenging assay. As shown in Figure 5b, DPPH formed a free radical with a maximum absorption peak at 517 nm. DAG containing one hydroxyl group had certain antioxidant properties, which reached a clearance rate of 13.62% at a concentration of 5 mg/mL. GTP with more hydroxyl groups has a higher radical scavenging ability through the resonance of delocalized electrons [37]. After the addition of GTP, the antioxidant activities of GTP-DAG-NLC and GTP-DAG-NLC-GEL were significantly improved, and the DPPH radicals scavenging rates reached 85.88% and 85.46%, respectively, when the concentration was 10 mg/mL.

#### 3.6.3. Anti-Glycation Activity

Metformin hydrochloride is one of the recognized hypoglycemic drugs, which can be used as a positive control group to evaluate the inhibition effect of hydrogel on the glycosylation reaction, which reflects the anti-glycation capacity of the hydrogel [38]. As shown in Figure 5c, the anti-glycation activities of GTP-DAG-NLC-GEL and DAG-NLC-GEL at different concentrations were higher than that of Metformin hydrochloride, and their inhibition rate of AGE were 89.72% and 92.11%, respectively, when the concentration was 10 mg/mL. GTP had no obvious anti-glycation properties; therefore, the anti-glycation performance of GTP-DAG-NLC-GEL loaded with GTP was significantly lower than that of DAG-NLC-GEL unloaded with GTP at the same concentration.

## 4. Conclusions

In this study, a novel material has been prepared by ultrasound emulsification to obtain GTP-loaded DAG-NLC-GEL, which exhibits a high encapsulation rate and good storage stability. Compared with GTP-DAG-NLC, GTP-DAG-NLC-GEL has higher viscosity and stability, indicating that the incorporation of hydrogel makes GTP-DAG-NLC suitable for the skin. The GTP release rate of GTP-DAG-NLC-GEL is lower than that of GTP-DAG-NLC, indicating that the addition of hydrogel slows the release of GTP so that GTP can stay on the skin surface for a longer time and prolong the efficacy. Combining the good UV absorption and antioxidant capacity of GTP with the good anti-glycation ability of DAG-NLC, GTP-DAG-NLC-GEL could be used as an excellent material for skin protection in cosmetics.

## Figures and Tables

**Table 1:** Particle size, PDI and zeta potential of the samples.

Sample Name	Size/nm	PDI	Zeta Potential/mV
GTP-DAG-NLC	141.77 ± 0.76	0.1091 ± 0.02729	−29.39 ± 0.42
DAG-NLC-GEL	292 ± 4.11	0.3007 ± 0.03833	−50.36 ± 0.86
GTP-DAG-NLC-GEL	299.07 ± 18.62	0.2991 ± 0.04091	−34.69 ± 0.87

**Table 2:** Comparison of physical properties of GTP-DAG-NLC-GEL with other nano-lipid carriers reported.

Sample Name	Size/nm	PDI	Zeta Potential/mV	EE/%	Reference
GTP-DAG-NLC-GEL	299.07 ± 18.62	0.2991 ± 0.04091	−34.69 ± 0.87	86.7	This work
NLC-DSPE-PEG2000-FA EGCG	359 ± 21	0.18 ± 0.01	−28 ± 1	85 ± 3	[22]
EGCG CTAB-LN	143.7 ± 0.450	0.24 ± 0.008	20.8 ± 0.896	-	[23]
EGCG-SLNs	115 ± 7	-	−55 ± 5	68.5	[24]
EGCG Liposome	120 ± 4	0.154 ± 0.028	−16.0 ± 5.3	58.94 ± 1.28	[25]
TP-lip	100.08 ± 2.33	0.22 ± 0.04	−30.85 ± 1.86	61.45 ± 0.23	[26]
PxL-EGCG	101.2 ± 13.4	0.396 ± 0.008	−43.0 ± 1.9	57.0 ± 4.5	[27]

**Table 3:** Particle size, PDI and zeta potential of GTP-DAG-NLC-GEL at different storage temperatures.

Temperature/°C	Storage Time/d	Size/nm	PDI	Zeta Value/mV
0~5	5	305.83 ± 2.31	0.3098 ± 0.03492	−33.45 ± 0.90
	10	307.01 ± 3.33	0.3244 ± 0.04512	−34.55 ± 0.74
	15	310.55 ± 2.41	0.3355 ± 0.00412	−35.12 ± 0.14
25	5	303.44 ± 5.61	0.3044 ± 0.05612	−33.44 ± 0.75
	10	311.31 ± 7.80	0.3541 ± 0.00846	−33.66 ± 1.26
	15	315.42 ± 5.12	0.3511 ± 0.00741	−34.12 ± 0.35

## Data Availability

The original contributions presented in this study are included in the article/supplementary material. Further inquiries can be directed to the corresponding author.

## Supplementary Materials

The following supporting information can be downloaded at: https://www.mdpi.com/article/10.3390/ma17246227/s1, Figure S1. (a) Particle size and PDI of six kinds of NLC. (b) Ultraviolet maps of six kinds of NLC. (c) DPPH free radical scavenging rate of six kinds of NLC. (d) Anti-saccharification properties of six kinds of NLC.
