# Supplementary material for: Preparation of Green Tea Polyphenol-Loaded Diacylglycerol Nanostructured Lipid Carrier Hydrogels and Their Activities Related to Skin Protection
Source: Materials (Basel). 2024 Dec 20;17(24):6227. doi: 10.3390/ma17246227 (PMC11676713; doi:10.3390/ma17246227)
Supplement: Supplementary file 1 [file materials-17-06227-s001.zip › materials-3355926-supplementary.pdf]

materials

Supporting Information

Preparation of green tea polyphenol-loaded diacylglycerol nanostructured lipid carrier hydrogels and their activities related to skin protection

Zhini Zhu [1,6], Qiu Xia [1,6], Xinxia Zhan [1], Wenyuan Li [1], Xuan He [2], Bo Wang [3], Qizhi Zhou [4], Jian Huang [5] and Yong Ye [1,*]

1 School of Chemistry and Chemical Engineering, South China University of Technology, Guangzhou, 510640, China
2 Ganzhou Hake Biotech Co., Ltd, Ganzhou, 341008, China;
3 Ganzhou Forestry Science Research Institute, Ganzhou 341000, China;
4 Hunan Singular Biotech Co., Ltd, Changsha 410329, China;
5 Jiangxi Ruijia Biotech Co., Ltd, Yichun 330899, China;
6 These authors contributed equally;
* Correspondence;
Tel.: +86-20-87110234

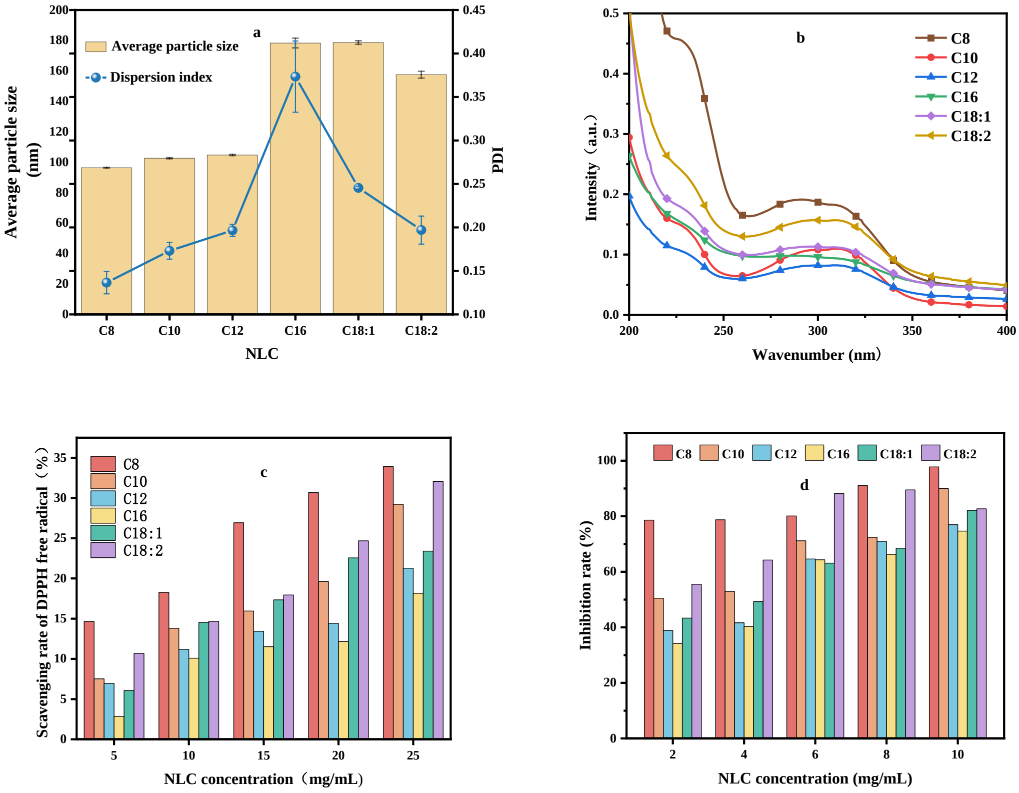

Figure S1. (a)Particle size and PDI of six kinds of NLC. (b)Ultraviolet maps of six kinds of NLC. (c) DPPH free radical scavenging rate of six kinds of NLC. (d) Anti-saccharification properties of six kinds of NLC.

In the initial phase of experimental screening, we investigated the properties of six materials: n-octanoic acid, n-decanoic acid, lauric acid, palmitic acid, oleic acid, and linoleic acid. As illustrated in Figure S1 (a), dioclylate NLC exhibits the smallest particle size

and the most stable system, as indicated by the particle size and PDI index. Figures S1 (b-d) present a comparative analysis of the sun protection, antioxidant, and anti-saccharification properties of the six types of NLC. The results demonstrate that C8-NLC possesses superior sun protection, antioxidant, and anti-saccharification properties. Therefore, diglycerin caprylate was selected as the raw material for NLC preparation.
